# Conflict of interest: use of pyrethroids and amidines against tsetse and ticks in zoonotic sleeping sickness endemic areas of Uganda

**DOI:** 10.1186/1756-3305-6-204

**Published:** 2013-07-10

**Authors:** Kevin Bardosh, Charles Waiswa, Susan C Welburn

**Affiliations:** 1 Centre of African Studies, School of Social and Political Science, College of Humanities and Social Science, The University of Edinburgh, 58 George Square, Edinburgh EH8 9LD, UK; 2 Department of Pharmacy, Clinical and Comparative Studies, School of Veterinary Medicine and Animal Resources, Makerere University, P.O Box 7062, Kampala, Uganda; 3 Division of Pathway Medicine and Centre for Infectious Diseases, School of Biomedical Sciences, College of Medicine and Veterinary Medicine, The University of Edinburgh, Chancellor’s Building, 49 Little France Crescent, Edinburgh EH16 4SB, UK

**Keywords:** Sleeping sickness, Trypanosomiasis, Tsetse, Insecticide, Veterinary drugs, Community-based disease control, Uganda

## Abstract

**Background:**

Caused by trypanosomes and transmitted by tsetse flies, Human African Trypanosomiasis and bovine trypanosomiasis remain endemic across much of rural Uganda where the major reservoir of acute human infection is cattle. Following elimination of trypanosomes by mass trypanocidal treatment, it is crucial that farmers regularly apply pyrethroid-based insecticides to cattle to sustain parasite reductions, which also protect against tick-borne diseases. The private veterinary market is divided between products only effective against ticks (amidines) and those effective against both ticks and tsetse (pyrethroids). This study explored insecticide sales, demand and use in four districts of Uganda where mass cattle treatments have been undertaken by the ‘Stamp Out Sleeping Sickness’ programme.

**Methods:**

A mixed-methods study was undertaken in Dokolo, Kaberamaido, Serere and Soroti districts of Uganda between September 2011 and February 2012. This included: focus groups in 40 villages, a livestock keeper survey (n = 495), a veterinary drug shop questionnaire (n = 74), participatory methods in six villages and numerous semi-structured interviews.

**Results:**

Although 70.5% of livestock keepers reportedly used insecticide each month during the rainy season, due to a variety of perceptions and practices nearly half used products only effective against ticks and not tsetse. Between 640 and 740 litres of insecticide were being sold monthly, covering an average of 53.7 cattle/km^2^. Sales were roughly divided between seven pyrethroid-based products and five products only effective against ticks. In the high-risk HAT district of Kaberamaido, almost double the volume of non-tsetse effective insecticide was being sold. Factors influencing insecticide choice included: disease knowledge, brand recognition, product price, half-life and mode of product action, product availability, and dissemination of information. Stakeholders considered market restriction of non-tsetse effective products the most effective way to increase pyrethroid use.

**Conclusions:**

Conflicts of interest between veterinary business and vector control were found to constrain sleeping sickness control. While a variety of strategies could increase pyrethroid use, regulation of the insecticide market could effectively double the number of treated cattle with little cost to government, donors or farmers. Such regulation is entirely consistent with the role of the state in a privatised veterinary system and should include a mitigation strategy against the potential development of tick resistance.

## Background

African trypanosomiasis refers to a group of parasitic diseases affecting people, livestock and wildlife transmitted by infected tsetse flies, found south of the Sahara and north of the Kalahari. African animal trypanosomiasis (AAT) or *nagana* is one of the most significant African livestock diseases with a major impact on cattle mortality and productivity [1-3]. Human African Trypanosomiasis (HAT) or sleeping sickness is caused by two related trypanosome sub-species, *T. b. gambiense* and *T. b. rhodesiense*, that are geographically separated by the Great Rift Valley. Gambian sleeping sickness is responsible for the majority of cases through human-tsetse transmission, but the zoonotic parasite (*T. b. rhodesiense*) involves a range of livestock and wildlife reservoirs in eastern and southern Africa [4]. Sleeping sickness is fatal if untreated and causes a significant human health burden in endemic foci where it is also often severely under-reported [5-9]. Largely under control in the late colonial era, HAT epidemics re-surfaced in the context of the ‘Great African Depression’ of the 1980s, but have been steadily declining since the late-1990s due to renewed global and national efforts [10-12].

With a ‘tsetse belt’ that runs from the southeast to the northwest of the country, over 70% of Uganda is infested with tsetse flies [13]. Bovine trypanosomiasis threatens approximately one-third of the national herd and is considered a major barrier to rural development [13]. Uganda is also the only country with both Rhodesian and Gambian forms of sleeping sickness; historically, the Gambian form has been confined to the northwest and the Rhodesian form to the southeast of the country [14].

**Figure 1:**
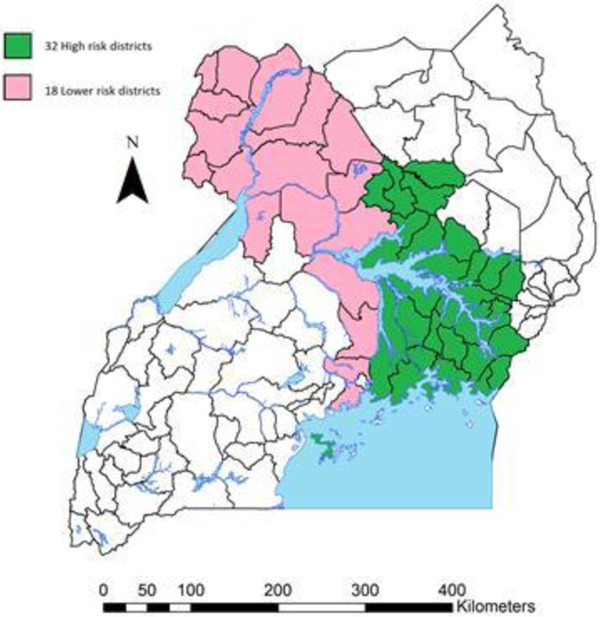
**Districts of Uganda that are either at high risk of Rhodesian sleeping sickness or are at risk of overlap between Gambian (chronic) and Rhodesian (acute) disease that should be considered as a priority for Acaricide Zoning.** There are 32 districts at high risk for Rhodesian sleeping sickness (approx. 2.6 million cattle). These are districts (highlighted in green) that have been historically affected by Rhodesian sleeping sickness and districts where humans are currently at risk of infection from the animal reservoir of infection (green). There are 18 further districts of Uganda where there have not yet been reported cases of Rhodesian sleeping sickness (approx. 1.8 million cattle) but which are at risk of immigration of acute disease from livestock movements, these include districts currently affected by Gambian sleeping sickness (pink).

Uganda has experienced a number of large-scale and smaller epidemics of sleeping sickness since the early 20^th^ century [14-16] and the disease now remains endemic. Thirty-two districts have been affected by Rhodesian (acute) sleeping sickness or are districts where humans are currently at risk from the disease. A further 18 districts are either affected by Gambian sleeping sickness (the chronic form of the disease) or are at significant risk of new migration of Rhodesian sleeping sickness (see Figure 1). From 2000 to 2009, Uganda reported 3,775 cases of Gambian sleeping sickness (of 170,486 total reported cases in Africa) and 2,848 cases of Rhodesian sleeping sickness (of 5,086 total reported cases) [12]. Vector control strategies applied in Uganda have included: mass land clearing, settlement relocation, game fences, elimination of wildlife and patient isolation used during the colonial era, to area-wide ground and aerial insecticidal spraying operations, centrally-coordinated and community-based tsetse trap campaigns, active case detection and treatment of livestock with veterinary drugs [10,15,17].

With changes in human population density, land-use and dramatically decreasing wildlife populations, the dominant reservoir-host for Rhodesian sleeping sickness has moved from wildlife to cattle in Uganda [18-21]. Since the late 1980s, the geographical range for Rhodesian HAT has increased from 13,820 to 34,843 km^2^ threatening to overlap with the *T. b. gambiense* foci [22]. Much of this spread has been caused by cattle movements, facilitated by both private traders and restocking programmes in Teso and Lango sub-regions (north of Lake Kyoga) which brought infected cattle into these naïve areas following the end of military conflict in the late 1990s and early 2000s [20-23]. This shift in reservoir dynamics has presented an opportunity to integrate bovine and zoonotic trypanosomiasis control through mass cattle treatments and farmer-driven vector control. Trypanocidal drugs eliminate all bovine trypanosomes including the zoonotic pathogen *Trypanosoma brucei rhodesiense* from the animal, while application of synthetic pyrethroid-based acaricide (insecticides that kill insects and arachnids, including both ticks and tsetse) turn cattle into moving tsetse traps or ‘live bait’ [9,24-26]. Since tsetse preferentially feed on the legs and belly of cattle, synthetic pyrethroids at dip concentration can be applied to only these sites on the animal - the so-called ‘restricted application protocol’ or RAP. RAP maintains efficacy while significantly reducing costs to farmers, minimising environmental impacts and minimising impact on endemic stability of tick-borne diseases [27,28].

By 2005, acute sleeping sickness had moved to within 150 km of the chronic disease foci. It was recognised that should the two forms of sleeping sickness merge then both diagnostic and treatment regimes would be entirely compromised, since the two parasites cannot be differentiated by microscopy alone and require different drug treatments [4]. In 2006 a public-private partnership, the Stamp Out Sleeping Sickness or SOS campaign (stampoutsleepingsickness.org) was established, specifically to halt disease spread and to prevent convergence of the two forms of sleeping sickness. Following a One Health approach, the plan was to remove the human infective parasites from cattle in five districts of northern Uganda in order to tackle an urgent human disease problem. The target was to treat 86% of cattle across the five at-risk districts and create a buffer zone between the *T. b. rhodesiense* and *T. b. gambiense* HAT foci [15]. Free mass cattle treatments were offered, using a single dose of trypanocide to remove both the human and animal infective parasites from cattle combined with three free monthly applications of insecticide using RAP to prevent reinfection of treated animals [29]. The intervention reduced the geographical range of *T. b. rhodesiense*, and reduced the prevalence of trypanosomes in cattle by 75% [23]. Driven by restocking efforts, continued movement of untreated cattle into Kaberamaido and Dokolo districts from other endemic areas led to the persistence of human cases and prompted a targeted re-treatment in 2008. To increase the use of pyrethroid acaricides by livestock keepers, the intervention then transitioned from a top-down mass treatment strategy to a bottom-up farmer and community-based approach [30,31]. Five recently graduated veterinary students (known as the 3 V Vets) were supported to provide community veterinary services, raise awareness, establish village-based spray teams and sell veterinary products, including the acaricide Vectocid© and the trypanocides Veriben© and Veridium©. The 3 V Vets successfully managed to create a diversified customer base in areas with little access to veterinary drugs and services, providing an additional source of pyrethroid-based acaricide. In late 2010, the SOS campaign was expanded to include Soroti and Serere districts, which involved conducting two free rounds of acaricide (synthethic pyrethroid) treatment followed by a combined trypanocidal and acaricide treatment and the establishment of six new 3 V Vets across these districts.

While mass cattle treatments significantly reduced both zoonotic and bovine trypanosomiasis [23,32], in order to sustain parasite control it is necessary for livestock keepers to use pyrethroid-based acaricides on a regular basis (to cope with tsetse re-invasion and the introduction of cattle from other endemic areas). A recent model has shown that using insecticide treated cattle (ITC) alone for zoonotic trypanosomiasis control in Uganda could eliminate the disease over time. If tsetse feed exclusively on cattle and people, as few as 20% of cattle need to be treated for the Basic Reproductive Rate of the disease to be less than one (*R*_*0*_ < 1) and for the disease to be eliminated [33]. Tsetse flies are highly susceptible to insecticides and ITC has been shown to offer the most economic trypanosomiasis control option in Uganda [34].

Long-term success and sustainability of zoonotic HAT control requires both treatment of the animal reservoir to eliminate the human infective pathogen plus sustained methods to prevent reinfection of these animals. Farmers in East Africa already use acaricides to target ticks and prevent tick-borne diseases such as anaplasmosis, babesiosis, cowdriosis and theileriosis, major causes of cattle mortality and morbidity [35-38]. Structural adjustment programmes (SAPs) in the late-1980s saw the withdrawal of state-subsidies in animal health, the privatisation of veterinary services and the liberalisation of the veterinary drug market [39]. Previous subsidisation of cattle dips started by many colonial governments fell into disuse. Most veterinary drugs are now sold by private agro-veterinary shops and a variety of trained and untrained animal health workers [35]. With the high cost of drugs many farmers self-treat, experiment with dilution rates and combine treatments, changing practices with seasons and income that may increase the risk of drug resistance [36-40]. Similarly, acaricides are a major private market in rural areas and both pyrethroid-based products (that are effective on both ticks and tsetse flies) and amidine-based products (effective on ticks but not on tsetse) are sold. Preventing reinfection of cattle with *T. b. rhodesiense* after mass cattle treatments in Uganda requires sustained monthly application of pyrethroid-based insecticides, which requires the provision of appropriate veterinary services, targeted education campaigns and access to acaricide at the village level.

This study examined the social dynamics of the veterinary drug market in four districts in northern and eastern Uganda where mass cattle treatments have been undertaken to control *T. b. rhodesiense*. The study aimed to: i) estimate the amount and type of acaricide sold in the area; ii) estimate the number of cattle sprayed each month; iii) explore the rationale for acaricide use and different application strategies; and iv) investigate the factors that influence supply and demand for different products by retailers and consumers.

## Methods

### Study area

The study area included two districts in eastern Uganda (Serere and Soroti in Teso sub-region) and two in northern Uganda (Dokolo in Lango sub-region and Kaberamaido in Teso). Geographically connected and part of the Lake Kyoga catchment area, the area covered approximately 6,107 km^2^ including over 773 km^2^ of open water and 562 km^2^ of extensive permanent and seasonal swampland that facilitates the survival of a moderate to low tsetse population [41]. The districts have reported over 1,300 cases of zoonotic sleeping sickness since 1998 while also being endemic for bovine trypanosomiasis. Most cases of sleeping sickness have been reported from Serere district (1998–2008) and Dokolo and Kaberamaido (2004-present) with only a few coming from what is now Soroti district (Serere was part of Soroti district until 2010).

A predominately rural society based on mixed crop-livestock farming and small-scale trades, the area is home to a mostly Christian population of the Lango, Iteso and Kumam ethnic groups with a population of over 600,000 people and over 400,000 cattle (Table 1). Farming activities revolve around a variety of different main crops (cassava, millet, sorghum, beans, maize and others) based on two growing seasons corresponding with the bimodal rains. Most of the area has an annual mean rainfall of 1,000 to 1,250 mm. Traditionally the first rain begins in March and ends in May/June while the second rain begins in September and ends in November/early December. There are intermittent light showers from June to August and the long dry season is from December to March. However, recent years have seen shifting rain patterns with heavy flooding during the second rainy season and increased rainfall between the two rainy seasons. Consistent with the rainfall pattern in 2011, this study considered the rainy season from March to late November/early December [41].

**Table 1:** District statistics

**District**	**Human population (2002 census data)**	**Cattle population (2008 census data)**	**Land area (Km**^**2**^**) (1995 census) **±	**Cattle density**
Dokolo	129,385	58,902	1,113ǂ	53/km^2^
Kaberamaido	131,650	76,109	1,354	56/km^2^
Soroti/Serere§	369,789	271,639	2,873	94.5/km^2^
TOTAL	630,824	406,645	5,340	76/km^2^

All figures are taken from the Ugandan Bureau of Statistics [41].

§ Data for Soroti and Serere districts were not available separately since Serere separated from Soroti in 2010.

± Land mass data excludes open water areas (269 km^2^ for Kaberamaido and 504 km^2^ for Soroti/Serere) but includes seasonal and permanent wetlands (144 km^2^ for Kaberamaido and 418 km^2^ for Soroti/Serere).

ǂWhile separate land/water area data for Dokolo district was not available, this has negligible impact on the calculation of cattle density since the district has relatively small open water and wetland areas.

As with much of northern Uganda, the Teso and Lango sub-regions experienced a variety of civil and military conflicts from the late-1980s to the mid-2000s, which has contributed to the high poverty rate found in the area [13]. Extensive cattle rustling by the north-western Karamojong ethnic group in the late 1980s devastated the Lango and Teso economy, an armed rebellion against the central government then lasted between 1986 and 1994 (The Teso War) which was then followed by the movement of the Lord’s Resistance Army (LRA) into these areas from the early to mid-2000s [42].

### Research methods

Research was conducted between September 2011 and February 2012. This involved a team of researchers (the lead-researcher, two translators and five research assistants) to accommodate the three linguistic groups in the area (Lango, Kumam and Ateso). A number of different qualitative and quantitative methods were applied [43,44] to investigate acaricide supply and understand acaricide demand and use.

#### Acaricide supply

To estimate the amount and type of acaricide sold in the area as well as to understand retailer practices, all veterinary shops across the four districts were identified and visited (n = 74) in November 2011. A detailed questionnaire with both open-ended and closed-ended questions was conducted with either the owner or attendant. Lasting between one and two hours, the questionnaire was divided into five sections: shop characteristics, sales information, customers and decision-making, knowledge of disease and shop linkages. While interviewees were asked to provide sales data on acaricides, in most cases records were unavailable and estimates for both the rainy and dry seasons had to be provided. This involved estimating the average amount and type of acaricide sold from December 2010 to March 2011 (dry season) as well as the months of September and November 2011 (two of nine months during the year with over 90 mm of rain) [41]. To verify the accuracy of the drug sale estimates a second short questionnaire was conducted with all shops either by telephone or in person between December 2011 and January 2012 (two of three months in 2011 with the lowest rainfall), which allowed for more accurate estimates for dry season sales. In the event of discrepancies between these estimates, an average from the two was then taken. Conducting questionnaires during business hours allowed for participant observation involving the interaction and level of information exchange between shop owners, attendants and livestock keepers. Unstructured and semi-structured interviews were also undertaken with these various groups in and outside of the veterinary shops.

A list of local community-based animal health workers and para-veterinarians was derived with the help of the drug shops and 26 semi-structured interviews were conducted over the study period. Interviews explored the practitioner’s work history, knowledge of disease, animal health practices, drug stock, drug use and sales practices. Numerous semi-structured interviews were also conducted throughout the study period with livestock extension workers, district officials (including all District Veterinary Officers, District Entomologists and District Medical Officers) and other key informants. Interviews explored past and current control strategies for human and animal trypanosomiasis and tick-borne diseases and the challenges and strengths of different potential future approaches.

#### Acaricide demand and use

A livestock-keeper survey on acaricide use (n = 495) was carried out to estimate the number of cattle sprayed each month, during the rainy season (October to November) in 56 villages across the four districts purposely selected for geographical variation. Participants were asked when they last sprayed their cattle with acaricide, the interval period between the most recent treatment, the treatment prior to that, the type of drug used and the reason for treatment. Dry season treatment interval estimates were considered unreliable and were excluded.

To explore the basis for acaricide use by livestock keepers, product preferences and different application strategies, focus groups were conducted in 40 villages (10 from each of the four districts) with separate male and female groups (between 6 to 15 participants). Villages were purposely selected, for geographical variation and different experiences of sleeping sickness, with help from district officials. Discussions took between one-and-a-half to two hours and included a specific focus on tsetse and trypanosomiasis control as well as a range of topics related to livelihoods, social organisation, veterinary care and human and animal health. To further explore local understanding and practices relevant to tsetse control, six villages with the highest number of reported zoonotic sleeping sickness cases in the area since 1998 were then selected and visited for between three to five days. Participatory methods (transect walks, natural resource use maps and seasonal calendars) as well as focus groups, interviews, direct observations and a household questionnaire (n = 94) were then conducted. These methods aimed to further clarify and investigate the interrelationships between livelihood patterns, cash spending habits, cattle management, veterinary services and drug use, knowledge of cattle diseases and sleeping sickness control.

### Ethical clearance and data analysis

Ethical clearance was obtained from the University of Edinburgh and verbal informed consent from every research participant following standard procedures [43,44]. Local leaders and district authorities were widely consulted and supportive of the study. Quantitative data were entered and analysed using Microsoft Office Excel© 2007 while qualitative data were entered into Microsoft Word© 2010 and analysed manually according to widely accepted methods of coding and memo writing [43,44].

## Results

### Acaricide supply

Veterinary drug shops were the main suppliers of acaricides in the area, procuring products in Kampala (Uganda’s capital) and selling them directly to farmers and to intermediary para-veterinarians, community-based animal health workers and government programmes. Seventy-four drug shops were identified in the area and the range of products, education of owners and attendants, and business practices differed widely (see Table 2). Veterinary shops in the area differed greatly, there were: a few large shops in Soroti town that acted as wholesalers to smaller shops; seven 3 V Vet shops supported by the SOS campaign; profitable and well-stocked shops in larger rural towns; shops located near weekly market sites; poorly stocked shops in more remote areas; and shops that opened sporadically without official licenses. While some areas reported a lack of shops, 80% of drug shops had been established within the last five years, which had undoubtedly increased the supply of acaricide in the area.

**Table 2:** Veterinary drug shop characteristics

**Number of shops per district**	
Dokolo	5
Kaberamaido	13
Serere	30
Soroti	26
**Shop age**	
Less than 1 year	9
Between 1 – 2 years	27
Between 3 – 5 years	23
Between 6 – 8 years	11
More than 9 years	4
**Education of shop owner**	
Veterinary degree	19
Diploma holder	33
Certificate holder	11
Community animal health worker	9
Para-veterinarian	2
Unknown	2
**Does the shop employ assistants?**	
Yes, with a diploma or certificate-level education	21
Yes, but no formal training	14
Yes, a para-vet	3
No	36
**Type of products/services available**	
Only veterinary drugs	18
Veterinary drugs and field services	42
Veterinary and agriculture drugs	11
Veterinary and agriculture drugs and field services	3
**Average estimated monthly shop revenue**	
Less than 1 million UgSH	13
Between 1 – 2 million UgSH	27
Between 2 – 3 million UgSH	19
Between 3 – 5 million UgSH	6
More than 5 million UgSH	6
Unknown	3

Disease ranking by shopkeepers consistently placed bovine trypanosomiasis, liverfluke and tick-borne diseases (anaplasmosis, theileriosis or East Coast Fever, and cowdriosis or Heartwater) as the most significant cattle diseases in the area. A total of 12 acaricide brands were sold: five products that are only effective against ticks (four amitraz products and one chlorfenvinphos) and seven pyrethroid-based products that are effective against both ticks and tsetse (see Table 3). The supply chain for acaricide products varied: 48 shops exclusively bought all products from Kampala; 16 imported all of the products into their respective district from both Kampala and another district within the study area (often from Soroti town); and 10 shops bought all acaricide only from other local shops both inside and outside their respective district. Acaricides were sold in bottles varying from 20, 50, 100 (the most popular), 250, 500 and 1000 ml as well as by individual ml.

**Table 3:** Acaricide brands marketed and sold

**Target vector**	**Brand name**	**Compound**	**Recommended dilution**	**Price range of 100 ml bottle (UgSH)**
Ticks	Amitix©	Amitraz	2 ml:1 L	5,000 – 8,000
	Milbitraz©	Amitraz	2 ml:1 L	7,000 - 9,000
	Norotraz©	Amitraz	2 ml:1 L	5,000 - 7,000
	Supona ©	Chlorfenvinphos	1 ml:2 L	6,000 – 7,000
	Tacktic©	Amitraz	2 ml:1 L	8,000 – 10,000
Ticks and tsetse	Alfapor©	Alpha-cypermethrin	1 ml:1 L	6,000 - 9,000
	Decatix©	Deltamethrin	1 ml:1 L	9,000 – 10,000
	Paratryn©	Cypermethrin	1 ml:1 L	10,000-12,000
	Sypertix©	Alpha-cypermethrin	1 ml:2 L	7,000 – 13,000
	Tsetse-tick©	Cypermethrin	1 ml:1 L	Not sold in 100 ml
	Cypermethrin-10 EC©	Cypermethrin	1 ml:1 L	8,000 - 10,000
	Vectocid©	Deltamethrin	1 ml:1 L	10,000- 15,000

The sale of acaricide varied by price, dilution rate and availability (see Table 3). Amidine-based products (which were predominately amitraz compounds) were cheaper to purchase ml for ml but these required double the concentration in application (following manufacturer recommendations). Pyrethroid products were more expensive but have a longer residual effect, target both ticks and tsetse and, if correctly diluted, were equivalent to the price of amitraz-based products in terms of application costs. While shopkeepers considered pyrethroid acaricides the more cost-effective and superior products, no shop in the area exclusively stocked them with most stocking a range of different amitraz and pyrethroid-based products.

The cheaper wholesale price of amitraz products (and the small amount of chlorfenvinphos sold) as well as tension between business interests and animal and public health concerns facilitated this disjunction. The cheaper price of amitraz allowed shops to free up more capital to purchase other products and it was common to find such shops with limited capital (often in more remote areas) stocking and selling greater quantities of amitraz at prices equivalent to pyrethroids found in more competitive areas. Despite attempts by some shops to disseminate information about the benefits of pyrethroids, even among the more educated owners who claimed to be dedicated to sleeping sickness control, amitraz compounds were actively stocked and sold. As one District Veterinary Officer (DVO) stated: *‘I have been very active in discouraging the use of amitraz and in every discussion with paravets I promote those good acaricides [pyrethroids] since farmers know it is better…but in business we have a liberalised economy and I have to go to the tune of customers…so I have to stock what people want, which is the cheapest acaricide….’* (Interview)

Since different customers demanded different products, shop owners claimed that it was necessary for them to stock products according to demand, despite their knowledge of product effectiveness. This was similarly the case for the many community-based animal health workers and para-veterinarians most of which stocked more than one type of acaricide to cater to customer demand. Although some supplied more pyrethroid insecticide, most sold significantly more amitraz in order to maintain a reputation for low prices. While a few larger shops and some community-based animal health workers and para-veterinarians were relatively active, efforts to educate and inform the client base on the benefits of pyrethroid use were generally perceived as time-consuming. Customer decision-making was driven by poverty, low educational status and ‘ignorance’ that were considered to drive customer preference for the cheapest available product. Many shop attendants were also de-motivated and poorly paid, which contributed to their lack of information exchange with customers about the benefits of pyrethroids.

### The acaricide market

**Figure 2:**
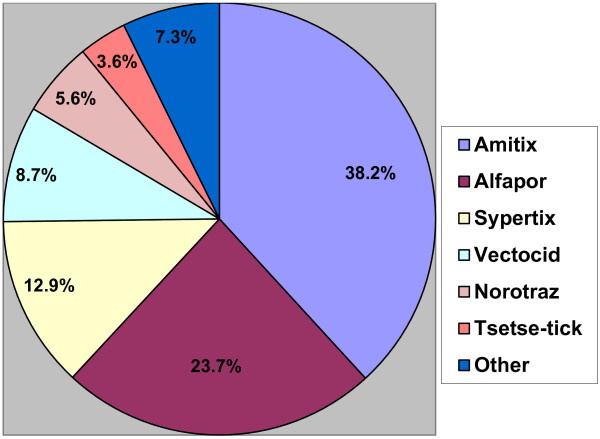
**The acaricide market divided by product sales.** Based on data from the 48 veterinary shops that exclusively imported products from Kampala. The data showed that an estimated 640.1 L of acaricide were sold during the rainy season each month in 2011.

Data from veterinary shops showed that a total of between 640.1 L (sold by the 48 shops exclusively acquiring products from Kampala) to 740.4 L of acaricide (including the 16 shops that sometimes acquired acaricides from other shops in the area) was reportedly sold per month during the rainy season. This included 51.4 to 52.2% pyrethroid-based and 47.8 to 48.6% non-tsetse effective products. While the amount of acaricide sold reduced significantly during the dry season (December to March) to between 404.8 to 470.6 L per month, the ratio between acaricide types remained similar (51.1% to 52.5% were pyrethroid products while 47.5% to 48.9% were non-tsetse effective). Based on the rainy season estimates from the 48 shops that exclusively acquired all products from Kampala, of the 12 acaricide brands sold, the most popular products (see Figure 2) included: Amitix© (an amitraz) which held 38.2% of the market share and the pyrethroid Alfapor© with 23.7% market share. These were followed by two pyrethroid acaricides, (Sypertix© (12.9%) and Vectocid© (8.7%)) and an amitraz product, Norotraz© (5.6%). Other acaricides in descending order of popularity included Tsetse-tick© (3.6%), Cypermethrin-10 EC© (1.9%), Milbitraz© (1.9%), Tacktic© (1.4%), Decatix© (1.1%), Supona© (0.7%) and Paratryn© (0.4%). The market share of the five leading products did not differ between the wet and dry seasons or the two different data sets (48 shops or 64 shops). Relative sales of the different brands also did not change significantly between the different data sets.

**Figure 3:**
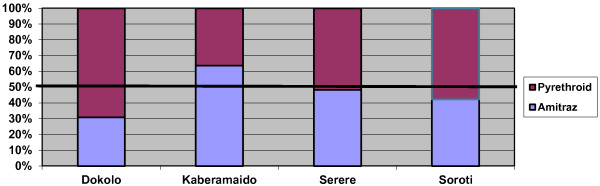
**The percentage of acaricide sold by product type per district.** (Based on monthly sales data from the 62 veterinary shops during the rainy season).

When aggregated, the ratio of amitraz (including the small amount of chlorfenvinphos sold) to pyrethroid products was roughly equal but this differed at district level (Figure 3). The larger amount of acaricide sold in Soroti compared to Serere district (Table 4) was partially due to a number of cheaper wholesalers in Soroti town that sold to smaller shops and community-based animal health workers in both Serere and Soroti. Likewise some acaricide was also sold from Kaberamaido district to Dokolo and neighbouring Amolatar district, mostly from a large livestock market on the border. While all other districts sold more pyrethroid-based acaricides in the rainy season, almost twice the amount of amitraz was sold in Kaberamaido during the rainy season (93.6 L compared to 53.5 L per month) (see Table 4 and Figure 3). While a small amount of this was used in the area that borders Dokolo, a few parishes in this same border region, have, since 2004 been the source of approximately 50 cases of Rhodesian sleeping sickness each year presenting to Lwala hospital and Dokolo Health Centre 4 [16]. Despite being knowledgeable about the problem of sleeping sickness and the role of cattle, the three veterinary drug shops located a few miles from Lwala Hospital (the largest HAT treatment centre in the area, located within these high risk parishes) were found to stock and sell significantly more amitraz products.

**Table 4:** District-level acaricide sales§

**Sales category**		**District**				
**Product type sold per month**	**Season**	**Dokolo**	**Kaberamaido**	**Serere**	**Soroti**	**Total**
Litres of pyrethroid	Dry season	21.4	29	52	125	240.4
	Rainy season	35.8	53.5	111.4	177.3	380.5
Litres of amitraz	Dry season	13.9	57.3	63	87.5	230.2
	Rainy season	16	93.6	104.2	129.8	359.9

§ Based on data from the 62 veterinary shops that imported all products into their respective districts but occasionally also purchased stock from other shops in the study area.

### Estimating the number of cattle sprayed

Estimating the number of cattle routinely sprayed each month, required triangulation. This involved focus groups with men (n = 40), a livestock-keeper survey (n = 495), a veterinary drug shop questionnaire (n = 74) and the many interviews and observations carried out over the six-month study period. Based on estimates for the rainy season, the livestock-keeper survey (n = 495) showed that 15.7% of respondents reported that they sprayed their cattle weekly, 21.5% fortnightly, 2.8% every 3 weeks, 24.3% every month, and 18.7% at irregular intervals, while 17% were found not to use acaricides at all. Of those livestock-keepers using acaricide, 35% reported using pyrethroids, 25% amitraz and 40% could not name the product. Making the conservative assumptions that those spraying irregularly did so every 3 months and those spraying every 3 weeks did so every month, an estimated 70.5% of these livestock-keepers reported spraying of their cattle at least once per month during the rainy season.

To assess the number of cattle sprayed we estimated, based on veterinary shop sales data, that an equal amount of pyrethroid and amitraz acaricide were used among the 40% of farmers that purported to use acaricide but could not recall the name of the product they used; according to data from the livestock keeper survey, 55% of acaricide usage was estimated to be pyrethroid-based and 45% amitraz-based per month. We also assumed that the interval between spraying was not influenced by the number of cattle a farmer owned. Based on data from the 2008 livestock census, for the four districts in the rainy reason, this would involve: 157,677 cattle sprayed at least once per month with a pyrethroid (38.5% of the cattle population); 129,008 cattle sprayed at least once per month with an amitraz (32%); 50,831 cattle would have received treatment 1 to 2 months previously that would now be ineffective (12.5%); and 68,975 (17%) would consistently be un-treated. Although some farmers heavily over-diluted and under-diluted acaricides, the majority did apply 1 ml to each animal with some applying 2 ml and very few 3 to 4 ml. Using the interval periods provided by the survey and a range of 1 to 2 ml, the total amount of acaricide required to spray 286,685 cattle each month during the wet season would be between 543.01 L to 1086.01 L, which fits within the range of 640.1 L to 740.4 L provided by the veterinary drug shop data.

To estimate the density of treated cattle we used the interval period provided by the livestock keeper survey (70.5% of cattle treated monthly) and the relative proportion of amitraz-to-pyrethroid sold in each district, based on the rainy season estimates from the 62 veterinary shops (Figure 3). An assumption was made that in general, if all acaricide sold in a district was used in that district (not always the case) and that spraying intervals during the rainy season remained similar between districts (observed to be the case) then an estimated 53.7 cattle/km^2^out of an average of 76 cattle/km^2^ across the 4 districts would be treated monthly in the rainy season: 27.6 cattle/km^2^ with pyrethroids and 26.1 cattle/km^2^ with amitraz products. However, there were significant differences between districts (Table 5) due to differences in cattle density (Table 1) and acaricide sales (Table 4). Cattle density also differed within individual districts. The south of Serere bordering Lake Kyoga has many more cattle than Soroti district or the northern part of Serere. Likewise the west of Kaberamaido (an area of high sleeping sickness risk) has fewer cattle per km^2^ than the eastern part of the district where few cases are reported. Given the significant difference between acaricide sales during the rainy and dry seasons (reductions of over 1/3 of sales), we can also assume that the density of treated cattle is reduced as farmers spray less and at longer intervals due to a lower perceived tick challenge.

**Table 5:** Density of cattle treated with acaricide types by district

**District**	**Pyrethroid-treated cattle**	**Amitraz-treated cattle**	**Total cattle treated**
Dokolo	25.7/km^2^	11.6/km^2^	37.3/km^2^
Kaberamaido	14.4/km^2^	25.2/km^2^	39.6/km^2^
Soroti/Serere	36.5/km^2^	30.2/km^2^	66.7/km^2^
Total area	27.6/km^2^	26.1/km^2^	53.7/km^2^

Interviews and focus groups showed that adhering to a prescribed spraying interval was a challenge for farmers due to competing interests and demands on time and money. Tick presence rather than a prescribed time period dictated the spraying interval. The number of farmers spraying monthly and the density of treated cattle are likely to be less than 70.5% and 53.7 cattle/km^2^. In focus group discussions with men, groups were asked to establish a percentage of how many farmers they believed sprayed at least once per month in the rainy season: 9 groups reported less than 40%; 18 groups between 40 to 60% and 13 groups more than 60%, giving an average of 55%. While there were observed variations in spraying intervals between different villages, the relative ratio of amitraz to pyrethroid products was consistent and not found to differ significantly. Application methods may also limit the efficacy of some of these treatments for both tick and tsetse control. Most farmers only targeted application to tick predilection sites, mainly on the ears, tail, udders/scrotum and hooves and some also applied product near the eyes, back, belly, thighs, legs and rear. To be effective against tsetse, application needs to cover tsetse predilection sites, the legs and belly of the animal. Application was also constrained by the ability of the livestock-keeper to restrain the animal (physically tied with ropes, use of cattle crushes or applied without any restraint) and the effectiveness of the spray equipment (hand pumps, ‘rwenzori’ plastic bottles with holes in the top, bundled leaves and grasses dipped into a bucket, and the use of hand pumps and, occasionally bucket or back sprayers).

### Factors influencing customer choice of acaricide brand

There were important differences in why livestock-keepers preferred specific acaricide brands (Table 6). Minor influences included: i) the colouring process during mixing (some less popular brands did not change colour in water, which made farmers suspicious of ‘being cheated’); ii) the effect on the animal’s coat (some products were believed to make the coat shine more than others); iii) the smell of the acaricide (some farmers expressed a preference for either weak or strong smelling products); iv) perceived side-effects on both cattle and people (pyrethroids were believed to be stronger, making cattle resistant to being sprayed and causing damage to people’s eyes and skin that could contribute to the development of ‘cancer’); and v) perceptions of tick resistance (amitraz was consistently believed to display the highest level of tick resistance). The more significant factors involved: disease knowledge; brand recognition; product price, half-life and mode of product action; availability; and information dissemination.

**Table 6:** Farmer perceptions found to influence acaricide choice

**Major factors**	**Minor factors**
Understanding of disease and vector	Dilution colour
Brand recognition	Effect on the animal’s coat
Price	Smell
Mode of product action	Perception of side effects
Product residual period	Perception of tick resistance
Availability	
Information dissemination	

### Understanding of vectors and disease

Basic knowledge about sleeping sickness was widespread in the area. However, this included the belief that HAT and AAT are transmitted by all blood feeding flies (biting flies, including stomoxys, were all locally perceived to be ‘tsetse flies’). There was awareness that HAT was a fatal disease with symptoms akin to malaria and HIV/AIDS that could be treated at certain health centres in the area. However, many people did not associate it with an animal reservoir, including a number of political leaders interviewed at the district and parish level and livestock-keepers in some of the most endemic villages. The household questionnaire (n = 94) conducted in six villages with the highest number of sleeping sickness cases in the area since 1998 showed that respondents identified removal of the invasive bush *Lantana camera* (96%), the use of tsetse traps (95%), avoidance of swampland (89%), not wearing blue clothing (71%), use of acaricide (54%) and active screening (24%) as the main methods of prevention.

For those who were aware of bovine trypanosomiasis, it was considered ‘cattle AIDS’: a chronic disease causing long-term production losses more often than acute signs of illness and death. The disease was most often associated with an animal looking for shady places, becoming thin and having a staring coat, which could be treated with ‘the drug that comes in the sacket’ (i.e. a trypanocide in a sachet). However, many farmers lacked a clear aetiological understanding of the disease and did not always relate it to ‘tsetse’. The livestock-keeper survey (n = 495) showed that only 21% of those who used acaricides claimed to spray for both ticks and tsetse flies, while the other 79% sprayed only for ticks despite the fact that most areas were infested with tsetse and suffering from various levels of trypanosomiasis. This was explained by the low tsetse challenge in the area, beliefs about disease risk (that acquiring human sleeping sickness was unlikely), the higher cost of pyrethroids and a belief that acaricides could only kill tsetse flies if the fly came into direct contact with the insecticide during spraying.

The link between the visible presence of tsetse and acaricide use was similarly drawn in relation to ticks. Understanding of tick-borne diseases involved three different general perceptions that were believed to be linked to educational status and geographical mobility: ticks were believed to cause specific diseases by transmitting pathogens; ticks caused many unknown diseases by either transmitting pathogens or by sucking blood; and ticks only caused physical morbidity that led to production losses. Socially frowned upon, the motivation for many, but not all, livestock keepers in spraying cattle was to remove ticks from the animal and not for the purpose of preventing the spread of tick-borne diseases.

### Brand recognition

An estimated 62% of sales were divided between two products: Amitix© (an amitraz) and Alfapor© (a synthetic pyrethroid). Both products are sold by the same manufacturer, have been on the market for over 15 years and resemble each other in their packaging. Individuals who knew the name of a product or selected products based on their packaging were very brand loyal. While Amitix© was the most well known acaricide, the packaging similarity with Alfapor© could facilitate a consumer shift from an amitraz to a pyrethroid and many livestock keepers considered Alfapor© a new and improved version of Amitix©. If a veterinary drug shop had sold out of Amitix©, shopkeepers found it easy to convince farmers to choose Alfapor© due to its close similarity in physical appearance and price, but it was more difficult to persuade farmers to purchase other products.

### Price and residual period

Although there were variations in price depending on the area, amitraz products were generally cheaper than pyrethroids (Table 3); however, if diluted and used according to recommendations these products are in fact more expensive for farmers since they require weekly application as opposed to the manufacturers recommended twice monthly application for pyrethroids. When focus groups were presented with this discrepancy, participants spoke at length about the difficulties of income generation for farmers, competing demands of different expenses on available income (for example, school fees), a general lack of financial planning and the high rates of illiteracy, drunkenness, domestic violence and other social problems that prevent development in the area. These discussions emphasised the fact that people, due to poverty, tended to gravitate towards the cheapest product option even when they knew it was not the most cost-effective. For instance, while many farmers reported that Vectocid© was the most effective product with the longest residual period (some farmers reported up to a month), the price deterred both veterinary shops from stocking it and customers from purchasing it. For those who chose pyrethroids (especially Vectocid©), their rationale was spoken about in terms of being ‘progressive’ and ‘modern’ and understanding the cost-benefit ratio of a lower dilution rate, longer residual period and, for some, protection against both tick-borne diseases and trypanosomiasis.

### Mode of action

The popularity of Amitix© had as much to do with price and brand recognition as with its mode of action. With many farmers motivated to spray only to remove ticks, perceived fast-acting acaricides were widely favoured over those products perceived to remove ticks at a slower rate. The majority of farmers, para-vets and veterinarians perceived amitraz-compounds as fast acting with tick removal/death occurring between 30 minutes to three hours and pyrethroid brands believed to be more slow acting with tick removal/death taking between one to three days.

### Availability and information dissemination

While the type of acaricide veterinary shops stocked certainly influenced the availability of specific brands, the willingness of drug shops and animal health workers to engage in information dissemination regarding the differences between acaricides also shaped consumer habits. One para-vet described what was confirmed in countless direct observations in veterinary shops: *“The names are there in the drug shops but most people don’t know, they are labelled but people don’t bother in checking or even many can’t read…they just heard ‘Amitix*©*’ so they say “Give me Amitix*©*” or they say “You give me an acaricide”…and then the shop owner tells him the prices without any other information about tsetse flies or dilution rates since they are not minding about education and he just takes the cheapest one.”* (Interview)

Basic factors around the motivation of the shop owner and attendant tended to influence whether they were active in telling farmers of the benefits of pyrethoids or would simply mention the price of different acaricides with farmers left to choose the cheapest product or the one they knew or had heard of.

### How to increase the use of pyrethroids?

Interviews and focus groups consistently explored how best to increase the use of pyrethroids and identified nine strategies (see Table 7). A number of these strategies have already been implemented in the area with poor results, including the formation of ad hoc spray groups and the rehabilitation of dips. While dip rehabilitation is actively sought by farmers (and is being supported in Kaberamaido district), experiences in the area show that it is unsustainable for a variety of reasons, these include: (i) high population density that creates challenges moving cattle to dips (ii) the high infrastructure and maintenance costs (iii) the need for user-fees and (iv) the desire of farmers to spray according to their own schedules. Sustainable implementation of village-level spray groups, as well as local bylaws to enforce mandatory spraying, are also not feasible given the lack of trust in local leadership structures and community dynamics in a post-conflict society. Farmers spray at different intervals, have money at different times, have different schedules and work plans and many would prefer to buy acaricides and apply it themselves. Ad hoc small scale attempts to collectively organise village-wide spraying for a small fee have consistently achieved low results. While strengthened community-based animal health worker networks have an important role to play in increasing pyrethroid use, service and supply of veterinary drugs (especially injectable drugs) present a more lucrative market than delivering spray services to the poorest farmers in the community. Animal health workers generally make most of their money (and spend most of their time) treating animals and see spray services as a side-business. Veterinary networks need to offer additional services to justify the costs of transport to remote areas. While there is a need for prioritisation of spray services in the community, this may require exploring new ways to diversify and offer a range of services to make business more viable.

**Table 7:** Interventions discussed by different stakeholders to increase pyrethroid use

**Possible intervention**	**Main strengths**	**Main weaknesses**
More sensitisation to communities	Education can address the many information gaps in disease transmission, the rationale for pyrethroid use and improve application strategies	Sensitisation has been on-going sporadically since 1998. Requires long-term engagement through repeated campaigns to significantly alter behaviour
		The nature of poverty in a subsistence-level economy will mean that the cheapest product will attract the most support
Creation of village bylaws	Creates collective ownership and a locally agreed enforcement strategy	Difficult to implement and sustain since the region is still recovering from decades of conflict and economic marginalisation
		Most communities are not willing or able to enforce spraying routines collectively
Encouragement of private sprayers	Increases supply of pyrethroids through the private market	Services are available in many areas but face challenges since farmers spray at different intervals
	Cattle can be organised every month for village-wide spraying	People support mass cattle treatments if they are free of charge or subsidised
	Strengthens access to veterinary services	Sprayer groups, such as those established through SOS, require incentives to reach the poorest communities and to make spray services a viable business as selling other veterinary services to farmers is seen to be more lucrative
	Provides local skills development and employment	
Cultivation of community spray groups	Group motivation facilitates compliance	Has been used in the past with little success
	Government/NGOs provide initial free inputs	Groups often fall apart due to insufficient local ownership
Rehabilitation of dips	Transfer of responsibility to government	Population density prevents/deters farmers from the movement of cattle
	Regular full body wash	User fees do not have local support
		People would rather spray according to their own schedule
Subsidise pyrethroid products	Equalises the perceived discrepancy in price (ml for ml) between pyrethroids and amitraz compounds	Requires continued outside financial support from public or private bodies
		Removal or alteration of subsidy can become a barrier to uptake and adoption
Educate veterinary shops and animal health workers	Relatively quick and can improve the skills of animal health workers	Shop owners and animal health workers already understand the benefits of pyrethroids but stock amitraz to meet customer demand
Government restriction of amitraz acaricides	Fastest solution that would avoid difficulties of facilitating behaviour change from farmers	In a liberalised economy, market restriction requires support from the central government, which could take a long time
Informal regulation of the market	Avoids the need for behaviour change and engaging in formal policy change	Requires political will at the district level

The most feasible and cost-effective solutions to getting more animals sprayed with pyrethroids were believed to relate to: (i) educating community-based animal health workers and veterinary drug shops; ii) provision of sensitisation campaigns targeted at livestock-keepers; (iii) subsidising pyrethroids; and (iv) some type of formal or informal government restriction of amitraz products. All of these solutions have merit. Veterinary shops in the area are well aware of sleeping sickness and the benefits of pyrethroid acaricides but owners and their staff continue to sell non-tsetse effective products due to established business norms and practices. Similarly, high poverty rates ensure that the cheapest products are the most popular with farmers. A number of sensitisation campaigns have been conducted in the area since 1998, including the SOS mass treatments and 3 V Vet activities that promoted the use of pyrethroids, and noticeable improvements over time in animal health practices were reported by district veterinary staff. While education on pyrethroid use should continue to form an important component of further programmes targeting sleeping sickness, participants believed that education alone had limitations both due to the nature of income generation and poverty in the area, as well as the need for prolonged dissemination to alter human behaviour. Similarly, while drug subsidies to support pyrethroid use could significantly increase the number of cattle sprayed, this would require external donor or private sector support and would need to align to a long-term plan with sustainable goals. If subsidies are provided and then subsequently removed or altered, any direct or perceived price rise can become a barrier to uptake and adoption.

Acaricide zoning (market restriction) consistently found support from community members and district officials as the most effective way to increase the use of pyrethroids. Zoning was believed to offer a quick solution to increasing the use of pyrethroid, since it would support the existing endogenous vector control practices of farmers. The main barrier to market restriction was believed to involve the need for support from policymakers and district enforcement since formal acaricide regulation in a liberalised economy would require endorsement from parliament and the national drug authorities. However, it was considered that district officials could organise an informal zoning strategy in areas where HAT and AAT co-exist by gathering together key stakeholders (i.e. all veterinary drug shops) and agreeing on a plan of action if sufficient local support was mobilised. Expressing the opinion of many, one village leader whose son had become mentally impaired after being treated for late-stage sleeping sickness stated: *“Burn those drugs that are causing us the problem of sleeping sickness…it is a serious problem here and just telling farmers like this will not change things since we are poor…you need to cut those drugs from the market…the government should stop the importation of those acaricides that don’t kill both ticks and tsetse flies to protect both cattle and human beings!”* (Interview)

## Discussion

Like other neglected diseases in Africa, the control of Rhodesian sleeping sickness has often been the remit of a public health system geared towards crisis management [15]. An ‘under-the-radar problem’, it is not only neglected by policymakers, donors and district officials but also by the same communities that suffer from it. In the absence of an epidemic, the local population, public health system and veterinary service are largely left to handle the problem on their own, which, given the competing interests at the district level and the multiplicity of challenges facing subsistence-level livestock keepers, rarely translates into effective prevention. This is similarly the case with the control of bovine trypanosomiasis, which also requires some degree of co-ordination and organisation to be effective. That not a single community-based tsetse trapping project has been maintained by community members after project funding ceased illustrates the difficulties of engaging farmers in resource-poor areas in what is essentially a public good, even when using relatively low-cost technology [45,46]. Engaging communities in locally acceptable solutions for neglected diseases like sleeping sickness requires thinking broadly about the multiplicity of factors that may mediate uptake and sustainability and exploring innovation pathways to what are complex problems embedded within specific social, cultural, ecological, political and economic realities.

Currently, the Ugandan government spends a significant but unpredictable amount of money on various control modalities through budgets from the central and district-level. Programmes actively promote the use of pyrethroids for tsetse and tick control as an avenue towards ‘healthier and wealthier communities’. In the mid-2000s, a large-scale $10 million dollar loan from the African Development Bank (ADB) with help from the International Atomic Energy Agency (IAEA) and the Pan African Tsetse and Trypanosomiasis Eradication Campaign (PATTEC) began the ‘Creation of Sustainable Tsetse and Trypanosomiasis Free Areas’ (STATFA) project. The project focussed mainly on animal trypanosomiasis and had planned for the release of sterile male flies supported by aerial spraying but instead implemented deployment of over 100,000 tsetse traps over 15 districts and treated over 450,000 cattle with pour-on acaricide [47]. No framework for sustaining pour-on insecticide use by farmers was put into place to ensure continued treatment after project activities ended.

In practice, acaricide use in Uganda has been largely ‘decentralised to the farmer’ but the boundaries between public and private good have become blurred. Government policy views livestock keeping as a ‘business’, where inputs (even to control certain, but not all infectious livestock and zoonotic diseases) must be paid for by the farmer while the infected patient suffering from zoonotic trypanosomiasis is treated by the public health system. Despite the rhetoric of the market, state involvement in animal health is justified in cases of market imperfections associated with public goods, externalities, information asymmetries and economies of scale as well as public health concerns [39,48]. Where the state should intervene, how and to what end remains an area of debate and these uncertainties have generally led to fragmented and somewhat unpredictable state policies governing the implementation of infectious livestock and zoonotic disease control in many developing countries [49].

Both HAT and AAT remain costly to livestock-keepers, infected patients and their families, and governments and donors [1-9,34]. With renewed attention being drawn to sleeping sickness through the increased profile of the neglected tropical diseases, the World Health Organisation (WHO) has targeted 2020 as an elimination date. The Stamp Out Sleeping Sickness campaign successfully reduced infection in livestock and has maintained the status quo between the two human forms of sleeping sickness. To upscale the SOS campaign and reap the dual benefits of HAT and AAT control farmers need to spray cattle on a regular basis with synthetic pyrethroid-based products. Additionally, in order to prevent the future spread of Rhodesian sleeping sickness through cattle movements and the potential overlap between the two human infective trypanosome parasites, mandatory trypanocidal treatment of cattle at livestock markets in endemic *T. b. rhodesiense* areas is needed [23].

The evidence presented here shows that acaricide use is a generally accepted technology in the study area. However, the acaricide market is almost equally divided between products effective against ticks and products effective against both ticks and tsetse flies. As a consequence nearly half of farmers were not using products that are beneficial for tsetse and trypanosomiasis control, even in villages with active cases of zoonotic sleeping sickness. The price difference between these two categories of products is very small (as little as $0.30 per 100 ml bottle). By simply restricting sales to pyrethroid-based products for tick and tsetse control in these HAT and AAT affected areas, the amount of pyrethroid applied to cattle on a monthly basis during the rainy season would almost double from an estimated 38.5% of cattle to 70.5% of the cattle population, a total of 286,882 out of 406,645 cattle (based on the 2008 census). This could potentially increase the density of pyrethroid-treated cattle from an estimated 27.6 cattle/km^2^ to 53.7/km^2^ throughout the four districts. Data from the veterinary shops showed Kaberamaido to be the only district that sells more amitraz than pyrethroid-based insecticide with an estimated cattle density of only 14.4 cattle/km^2^ treated with pyrethroid in comparison to 25.2 cattle/km^2^ treated with amitraz. This district also reported the most cases of zoonotic trypanosomiasis in Uganda in 2011, while the other three districts included in this study reported very few cases.

During the British colonial era in Uganda, cattle dips were zoned with different acaricides being used every few years in different parts of the country in order to prevent the development of tick resistance. During this study, many farmers and veterinarians commented on the pervasiveness of tick resistance to amitraz compounds (especially Amitix©) while a few also commented on resistance to pyrethroids, notably Alfapor©. Both of these products were found to be the most widely sold in the area with 62% of the market share. Of the 74 veterinary shops in the area, 80% of them had opened within the last five years and interviews consistently emphasised that acaricide usage had increased dramatically within that time. Together with increased agricultural production, district medics and veterinarians in Serere considered that acaricide usage was contributing to the decrease in sleeping sickness cases since the mid-2000s. As farmers increase acaricide use, studies are needed to explore any possible environmental impacts, effects on tick-borne disease epidemiology (notably, endemic stability) and, importantly, monitor the emergence of tick resistance to both amitraz and pyrethroid compounds. While increased pyrethroid use will have significant human health and animal health benefits, it is unlikely that acaricide zoning alone would further escalate any of these possible risks, especially as the current strategy of the district veterinary office, as well as the national authorities is to promote more, and not less, acaricide use. One way to mitigate these impacts is to promote the use of the restricted application protocol or RAP [24-28] and/or to have alternating products introduced into the area every few years.

There are plans to build on the SOS model by implementing the large-scale use of intensive mass cattle treatments (trypanocides and RAP) to sustainably reduce Rhodesian sleeping sickness across all 32 high-risk districts in Uganda and to promote RAP adoption in the lower risk districts (see Figure 1). This will reduce human disease burden, remove the risk of disease convergence and roll out sustainable farmer based AAT and tick control. An initial three-year mass cattle treatment programme – injection and spray– is proposed to quickly reduce human infective parasite prevalence in cattle. The gains achieved through mass treatments would be sustained by community-based spray teams, tasked to deliver on-going insecticide treatment to cattle in the high-risk areas. Interventions need to be implemented rapidly and at scale. The impact of the proposed intervention will be evaluated in terms of effective delivery of the mass treatment programme in years 1–3 and the reduction in the human infective parasite prevalence rate in cattle in years 4–8. It is proposed that the intervention be delivered through a Development Impact Partnership [50]. In order to sustain gains achieved through mass treatment, community-based networks of insecticide sprayers established during the mass treatment phase will aim for continued restricted application treatment of 15 - 20% of cattle on a monthly basis, which will also provide local employment and strengthen animal health provision. To optimise farmer-driven tsetse control, restriction on the sale of non-tsetse effective acaricide products in these areas should be implemented. Based on results presented here, this would double the amount of cattle being sprayed monthly for tsetse control with significant benefits for human health, livestock health and agricultural production in some of the poorest areas of Uganda.

## Conclusion

Routine application of acaricide to cattle is a cost-effective control method that farmers can adopt for both tsetse and tick control in much of Africa and is crucial to the long-term success of mass trypanocidal treatment for the control of Rhodesian sleeping sickness in Uganda. However, not all acaricide products on the private veterinary market are effective on tsetse since many are only effective on ticks. In areas with a high risk of zoonotic sleeping sickness in Uganda and/or bovine trypanosomiasis, market restriction of non-tsetse effective products has the potential to double the number of animals acting as ‘live bait’ for tsetse. This would require little direct cost to government, donors or farmers since it builds upon existing practices. The main barriers are with policy implementation and political will, which have been known to play a major role in mediating the effectiveness of tsetse and trypanosomiasis control. While formal government acaricide zoning is needed to create the necessary policy environment for enforcement, by involving key stakeholders at the district-level, informal zoning could facilitate the increase in pyrethroid use much more quickly in areas with active acute sleeping sickness cases. At this time, however, the lack of a policy framework to support farmer practices allows the ‘invisible hand’ of the free market to guide supply and demand to the detriment of tsetse control and hence animal and human health. While not a panacea, market restriction of non-tsetse effective acaricide products should be considered in all trypanosomiasis endemic areas of Uganda in order to optimise existing farmer-driven vector control and support future disease elimination efforts for *T. b. rhodesiense*.

## Competing interests

The authors declare they have no competing interests and the sponsors had no role in the study design, data collection and analysis, decision to publish,or preparation of the manuscript.

## Authors’ contributions

Conceived and designed the experiment: KB, CW, SCW. Performed the experiment: KB. Analyzed the data: KB Contributed reagents/materials/analysis tools: KB, SCW. Wrote the paper: KB, SCW. All authors read and approved the final manuscript.

## References

[B1] Swallow B Impacts of Trypanosomiasis on African Agriculture 2000 Rome: Food and Agriculture Organisation

[B2] Kristjanson P Swallow B Rowlands G Kruska R deLeeuw P Measuring the costs of African animal trypanosomiasis, the potential benefits of control and returns to research Agric Syst 1999 59 79 98 10.1016/S0308-521X(98)00086-9

[B3] Shaw A Maudlin I, Holmes P, Miles M Economics of African Trypanosomiasis The Trypanosomiases 2004 Cambridge: CABI Publishing 369 402

[B4] Fèvre E Picozzi K Jannin J Welburn S Maudlin I Human African Trypanosomiasis: epidemiology and control Adv Parasitol 2006 61 167 221 16735165 10.1016/S0065-308X(05)61005-6

[B5] Odiit M Coleman P Liu W McDermott J Fèvre E Quantifying the level of under-detection of *Trypanosoma brucei rhodesiense* sleeping sickness cases Trop Med Int Health 2005 10 840 849 10.1111/j.1365-3156.2005.01470.x 16135190

[B6] Odiit M Shaw A Welburn S Fevre E Coleman P Assessing the pattern of health-seeking behaviour and awareness among sleeping sickness patients in eastern Uganda Ann Trop Med Parasitol 2004 98 339 348 10.1179/000349804225003389 15228715

[B7] Matemba LE Fèvre EM Kibona SN Picozzi K Cleaveland S Quantifying the burden of rhodesiense sleeping sickness in Urambo District, Tanzania PLoS Negl Trop Dis 2010 4 11 10.1371/journal.pntd.0000868 PMC2970539 21072230

[B8] Fèvre EM Wissmann BV Welburn SC Lutumba P The burden of human African Trypanosomiasis PLoS Negl Trop Dis 2008 2 12 10.1371/journal.pntd.0000333 PMC2602597 19104653

[B9] Lutumba P Makieya E Shaw A Meheus F Boelaert M Human African trypanosomiasis in a rural community, Democratic Republic of Congo Emerg Infect Dis 2007 13 248 254 10.3201/eid1302.060075 17479887 PMC2725878

[B10] Maudlin I African trypanosomiasis Ann Trop Med Parasitol 2006 100 8 679 701 10.1179/136485906X112211 17227648

[B11] Simarro PP Diarra A Ruiz Postigo JA Franco JR Jannin JG The Human African Trypanosomiasis Control and Surveillance Programme of the World Health Organization 2000–2009: the way forward PLoS Negl Trop Dis 2011 5 2 10.1371/journal.pntd.0001007 PMC3042999 21364972

[B12] Simarro PP Cecchi G Paone M Franco JR The Atlas of human African trypanosomiasis: a contribution to global mapping of neglected tropical diseases Int J Health Geogr 2010 9 57 10.1186/1476-072X-9-57 21040555 PMC2988709

[B13] Ministry of Agriculture Animal Industry and Fisheries, Uganda; Uganda Bureau of Statistics; Food and Agriculture; Organization of the United Nations; International Livestock Research Institute; and World Resources Institute: Mapping a Better Future: Spatial Analysis and Pro-Poor Livestock Strategies in Uganda 2010 Washington, DC and Kampala: World Resources Institute

[B14] Fèvre EM Coleman PG Welburn SC Maudlin I Reanalyzing the 1900–1920 sleeping sickness epidemic in Uganda Emerg Infect Dis 2004 10 567 573 10.3201/eid1003.020626 15200843

[B15] Welburn S Coleman P Maudlin I Fevre E Odiit M Crisis, what crisis? Control of Rhodesian sleeping sickness Trends Parasitol 2006 22 123 128 10.1016/j.pt.2006.01.011 16458071

[B16] Batchelor N Atkinson PM Gething PW Picozzi K Fèvre E Kakembo A Welburn S Spatial predictions of Human African Trypanosomiasis prevalence in Kaberamaido and Dokolo, two newly affected districts of Uganda PLoS Negl Trop Dis 2009 3 12 10.1371/journal.pntd.0000563 PMC2788694 20016846

[B17] Hoppe K Lords of the fly: sleeping sickness control in British East Africa, 1900–1960 2003 Praeger: Connecticut and London

[B18] Hide G History of sleeping sickness in East Africa Clin Microbiol Rev 1999 12 112 125 9880477 10.1128/cmr.12.1.112 PMC88909

[B19] Hide G Tait A Maudlin I Welburn SC The origins, dynamics and generation of *Trypanosoma brucei rhodesiense* epidemics in East Africa Parasitol Today 1996 12 50 55 10.1016/0169-4758(96)80654-5 15275254

[B20] Fèvre E Coleman P Odiit M Magona J Welburn S The origins of a new *Trypanosoma brucei rhodesiense* sleeping sickness outbreak in eastern Uganda Lancet 2001 358 625 628 10.1016/S0140-6736(01)05778-6 11530149

[B21] Fèvre E Picozzi K Fyfe J Waiswa C Odiit M A burgeoning epidemic of sleeping sickness in Uganda Lancet 2005 366 745 747 10.1016/S0140-6736(05)67179-6 16125592

[B22] Picozzi K Fevre E Odiit M Carrington M Eisler M Sleeping sickness in Uganda: a thin line between two fatal diseases BMJ 2005 331 1238 1241 10.1136/bmj.331.7527.1238 16308383 PMC1289320

[B23] Selby R Limiting the northerly advance of Trypanosoma brucei rhodesiense in post conflict Uganda 2011 PhD thesis: University of Edinburgh

[B24] Hargrove J Omolo S Msalilwa J Fox B Insecticide-treated cattle for tsetse control: the power and the problems Med Vet Entomol 2000 14 123 130 10.1046/j.1365-2915.2000.00226.x 10872856

[B25] Hargrove J Tsetse eradication: sufficiency, necessity and desirability 2003 Edinburgh: DFID Animal Health Programme http://r4d.dfid.gov.uk/PDF/Outputs/RLAHtsetse_Erad.pdf

[B26] Hargrove J Torr S Kindness H Insecticide-treated cattle against tsetse (Diptera: Glossinidae): what governs success? Bull Ent Res 2003 93 203 217 10.1079/BER2003234 12762862

[B27] Torr S Maudlin I Vale G Less is more: restricted application of insecticide to cattle to improve the cost and efficacy of tsetse control Med Vet Entomol 2007 21 53 64 10.1111/j.1365-2915.2006.00657.x 17373947

[B28] Bourn D Grant I Shaw A Torr S Cheap and safe tsetse control for livestock production and mixed farming in Africa Asp Appl Biol 2005 75 1 12

[B29] Stamp Out Sleeping Sicknesshttp://www.stampoutsleepingsickness.com/

[B30] Morton J The innovation trajectory of sleeping sickness control in Uganda Research knowledge in its context http://www.researchintouse.com/resources/riu10discuss08ssickcntrl-ug.pdf

[B31] Bardosh K The use of private veterinarians in tsetse and trypanosomiasis control: The Stamp Out Sleeping Sickness Campaign in Northern Uganda 2010 MSC thesis: University of Edinburgh

[B32] Fyfe J The impact and outcomes of recent control interventions against trypanosomiasis in eastern Uganda 2007 PhD thesis: University of Edinburgh

[B33] Hargrove JW Ouifki R Kajunguri D Vale GA Torr SJ Modelling the Control of Trypanosomiasis Using Trypanocides or Insecticide-Treated Livestock PLoS Negl Trop Dis 2012 6 5 10.1371/journal.pntd.0001615 PMC3352824 22616017

[B34] Shaw A Torr S Waiswa C Cecchi G Wint GR Estimating the costs of tsetse control options: an example for Uganda Prev Vet Med 2013 110 290 303 10.1016/j.prevetmed.2012.12.014 23453892

[B35] Bett B Machila N Gathura P McDermott J Eisler M Characteristics of shops selling veterinary medicines in a tsetse-infested area of Kenya Prev Vet Med 2004 63 29 38 10.1016/j.prevetmed.2004.02.004 15099714

[B36] Mugabi K Mugisha A Ocaido O Socio-economic factors influencing the use of acaricides on livestock: a case study of the pastoralist communities of Nakasongola District, Central Uganda Trop Anim Health Prod 2010 42 131 136 10.1007/s11250-009-9396-6 19543802

[B37] Mugisha A McLeod A Percy R Strategies, effectiveness and rationale of vector-borne disease control in the pastoralist system of South-western Uganda Trop Anim Health Prod 2005 6 479 489 16248219 10.1007/s11250-005-2174-1

[B38] Mugisha A Mcleod A Percy R Socio-economic factors influencing control of vector-borne diseases in the pastoralist system of south-western Uganda Trop Anim Health Prod 2008 40 287 297 10.1007/s11250-007-9093-2 18557192

[B39] Leonard D Africa’s changing markets for health and veterinarian services: the new institutional issues 2000 London: MacMillian Press

[B40] Geerts S Holmes P Diall O Eisler M African bovine trypanosomiasis: the problem of drug resistance Trends Parasitol 2001 17 25 28 10.1016/S1471-4922(00)01827-4 11137737

[B41] 2012 Statistical Abstract http://www.ubos.org/onlinefiles/uploads/ubos/pdf%20documents/abstracts/2012%20Statistical%20Abstract.pdf

[B42] Jones B Beyond the state in rural Uganda 2009 Edinburgh: Edinburgh University Press for the International African Institute 199

[B43] Bourgeault I Dingwall R de Vries R Sage Handbook on Qualitative Health Research 2010 London: Sage Publications

[B44] Padgett D Qualitative and Mixed Methods in Public Health 2012 Thousand Oaks, USA: Sage Publications

[B45] Brightwell B Dransfield B Maudlin I Stevenson P Shaw A Reality vs. rhetoric- a survey and evaluation of tsetse control in East Africa Agriculture and Human Values 2001 18 219 233 10.1023/A:1011131826919

[B46] Dransfield R Brightwell R Maudlin I, Holmes P, Miles M Community participation in tsetse control: the principles, potential and practice The Trypanosomiases 2004 Cambridge: CABI Publishing 533 546

[B47] African Development Bank Uganda: Creation of sustainable tsetse and trypanosomiasis free areas in East and West Africa: Project completion report http://www.afdb.org/fileadmin/uploads/afdb/Documents/Project-and-Operations/Uganda%20-%20Creation%20of%20Sustainable%20Tsetse%20and%20Trypanosomiasis%20Free%20Areas%20in%20East%20and%20West%20Africa%20-%20Project%20Completion%20Report%20%28PCR%29.pdf

[B48] Umali D Feder G Haan C Animal health services: finding the right balance between public and private delivery The World Bank Research Observer 1994 9 71 96 10.1093/wbro/9.1.71

[B49] Scoones I Wolmer W Livestock, disease, trade and markets: policy choices for the livestock sector in Africa Sussex: Institute of Development Studies, University of Sussex ftp://ftp.fao.org/docrep/nonfao/LEAD/af853e/af853e00.pdf

[B50] Case Study 1 Reduction of Rhodesian sleeping sickness in Uganda, Development impact bond working group report, 2013, 46–53. Centre for International Development and Social Financehttp://www.socialfinance.org.uk/resources/social-finance/dib-working-group-report-consultation-draft

